# Editorial: Frontiers in Chemistry: 10 years anniversary

**DOI:** 10.3389/fchem.2025.1572259

**Published:** 2025-02-20

**Authors:** Steven L. Suib

**Affiliations:** Department of Chemistry, University of Connecticut, Storrs, CT, United States

**Keywords:** chemistry, research, anniversary, 10th year, review

We are thrilled to celebrate a significant milestone for *Frontiers in Chemistry*—our 10-year anniversary! To commemorate this remarkable occasion, we have meticulously curated an extraordinary anniversary collection, featuring a series of groundbreaking papers, including insightful contributions from our esteemed Specialty Chief Editors. These works illuminate the cutting-edge frontiers and diverse domains of modern chemistry. With this Anniversary Research Topic, our aim is to present both new research and reviews of the most exciting areas of current chemical research.

Pannico et al. have studied the thermal oxidation of flexible epoxy resins using *in situ* FTIR. We report on an *in situ* FTIR study of the thermo-oxidative degradation of a flexible epoxy resin. A unique feature was the normal coordinate analysis used to help interpret the vibrational data. The structure of such resins is a network as determined by using two-dimensional correlation spectroscopy. This paper is an excellent combination of spectroscopy, modeling, kinetics, and synthesis.

A team led by Félix et al. has also focused on excellent characterization of luminescent cage-like tetranuclear silsesquioxanes. Synthesis, X-ray diffraction, magnetism, and luminescence studies of single crystals were done. Such systems are a series of seven-coordinate lanthanides that have a one-capped trigonal prismatic structure. Luminescence was sensitized by acetylacetonate ligands when excitation was in the UV and visible spectral regions. Computational modeling of intramolecular energy transfer and multiphonon rates was used to study thermal interactions in mixed lanthanide ions. Such research has led to the development of luminescent thermometers. These optical sensing materials may lead to numerous other applications.

Xu and Biczysko have used vibrational spectroscopy with the James Webb Space Telescope to investigate molecules in interstellar space. This truly unique work focused on cyano-based derivatives of aromatics. A key molecule that was identified was benzonitrile. This molecule was used as a model via computational modeling to try to observe similar but heretofore unknown molecules. Anharmonic simulations of infrared data were compared to experimental Fourier transform infrared data. Their calculations provided excellent math to experimental data, suggesting that this methodology could provide reliable predictions of vibrational data for a large variety of cyano-based interstellar species.

Gavara et al. have reviewed the use of Covalent organic frameworks (COFs) as chromatographic solid stationary phases. They suggest that COFs have various structural motifs with different chemical and physical properties that allow these separations. In their review, the types of ideas that have been used to lead to chromatographic applications primarily as stationary phases. They have also suggested that this research is novel, still in development, and may lead to other types of chromatographic applications to solve different analytical problems.

The above characterization studies provide a wealth of information regarding the use of characterization methods to study a wide variety of materials for a diverse set of applications. Indeed, the development of new and modified methods both *ex situ* and *in situ* are the focus of many research groups around the world. New instrumentation, new sample holders, combined methods, and studies of unique systems have all revolutionized the field of chemistry.

Another area of recent and continuing interest is catalysis. Garg et al.’s group has emphasized and reviewed the significance of asymmetric catalysis for efficient synthesis of enantiopure chiral systems. Transition metals are key to transformations with chiral ligands that are important in this type of catalysis. Phosphines and carbenes are types of species used to form chiral C-x and C-C bonds. This leads to specific control of stereochemistry. Photocatalytic, electrocatalytic, and biocatalytic systems have been covered in this review. Sustainability, atom-efficiency, environmentally friendly, and versatility properties are all important in this area of research. Enantioselective organocatalytic reactions have also been covered. The novelty of flow methods in asymmetric catalysis was mentioned as transformative. Hybrid approaches with different strategies that are synergistic have been incorporated to attack challenging problems.

Ali and Visser have contributed a paper on catalytic divergencies in the mechanism of L-arginine hydroxylating nonheme iron enzymes. Such enzymes can be important in the biosynthesis of natural products. Their focus has been on mechanistic studies of L-Arg-activating nonheme iron dioxygenases. As with many of the above papers in this anniversary Research Topic, computational modeling, molecular dynamics, and quantum mechanical methods have been used to predict a variety of properties such as electrostatics and electronics. These studies suggest that changes in electric fields and electric dipole moments can strengthen or weaken C−H bonds. This can lead to unique reactivity of such enzymes and their novel reaction pathways.

Tsvetkov et al. have studied Brucellosis which is a dangerous disease caused by bacteria. This work aimed at new methods for diagnosing brucellosis. Specific methodologies were summarized and the authors pointed out that false positives are observed. Synthetic oligosaccharides can improve the reliability of these tests. This epitope is characterized by an α-(1→3)-linkage between d-perosamine units and is unique to *Brucella*. They developed a set of four protected oligosaccharides of two trisaccharides and a tetrasaccharide to improve diagnostic reliability. They plan to extend such an approach to both animals and humans, and to developing potential vaccines.

The work of Chan et al. concerns fragment-based drug discovery for treating central nervous system disorders. This approach uses screening of libraries of thousands of small molecular fragments. This approach allows a more strategic starting point than typical high throughput screening methods. Advantages of using such fragments include compactness, good binding affinities, important in drug candidate development. This method can allow for precise modifications to improve activity and pharmacokinetics. There has bene commercial success of this methodology and has promise for fast drug discovery and development. This review suggests how this approach can be integrated into drug discovery.

Otvos and Wade have reviewed big peptide drugs. Designer peptide drugs developed by biotechnology companies are injectable and have been useful for orphan and small volume diseases like type 2 diabetes. Successful oral doses have been used for chronic constipation. Careful choices of targets, modification of intrachain and terminal amino acids, correct conjugation for stabilizing enhancers, unique delivery methods, and marketing strategies focused on patients have been successfully used to create useful treatments. Improvement in peptide drug modification and formulation has led to significant growth and acceptance of designer peptide drugs as desirable alternatives to small molecule compounds.

Nulakani and Ali have studied the chemical kinetics of aminomethanol (NH_2_CH_2_OH) systems for insight into O.H and O_2_ photo-oxidation reactions. Aminomethanol has been released into the atmosphere via burning of biomass and from other pathways. The chemical kinetics of aminomethanol in relation to hydroxyl radicals have been investigated using a variety of computational modeling methods and studies. Computational modeling as a sole source of analysis as well as in conjunction with experimental methods has been another theme that links many of the papers in this Anniversary Research Topic. Activation energies for these reactions, rate constants, and branching paths of radical species were pursued. They also studied detrimental effects of formamide. Formamide can react with hydroxyl radicals to form toxic materials like HNCO.

Another original article by Tang and He concerns ways to remove arsenic from nickel-based systems. Thermodynamic calculations and studies of roasting and reduction methods were described. Roasting under oxidizing conditions was not productive. However, sulfur was used as a reducing agent and this reacted with arsenic to form arsenic sulfide, which is volatile and can be removed. Use of FeS under oxidizing conditions was not as promising due to reactions of nickel with arsenic at high temperatures. On the other hand, use of FeS under reducing conditions appears to be the best method that was studied here for removal of arsenic.

The above articles are excellent reviews and original research that highlight many of the current and new directions being used in the field of chemistry. Some unique underlying themes include advanced characterization, new synthetic strategies, and the use of computational modeling to understand all these types of systems described above.

